# Removal of Cationic and Anionic Dyes from Aqueous Solution with Activated Biocarbons Obtained from Black Chokeberry Seeds

**DOI:** 10.3390/ma19040707

**Published:** 2026-02-12

**Authors:** Paulina Marciniak, Marlena Groszek, Małgorzata Wiśniewska, Zhanat Idrisheva, Togzhan Toktaganov, Piotr Nowicki

**Affiliations:** 1 Department of Applied Chemistry, Faculty of Chemistry, Adam Mickiewicz University in Poznań, Uniwersytetu Poznańskiego 8, 61-614 Poznań, Poland; paulina.marciniak@opoczta.pl; 2 Department of Radiochemistry and Environmental Chemistry, Institute of Chemical Sciences, Faculty of Chemistry, Maria Curie-Skłodowska University in Lublin, M. Curie-Skłodowska Sq. 3, 20-031 Lublin, Poland; marlena.geca@wp.pl; 3 School of Geosciences, D. Serikbayev East Kazakhstan Technical University, 19, Serikbayev Str., Ust-Kamenogorsk 070000, Kazakhstan; tokta94@gmail.com

**Keywords:** black chokeberry seeds, waste biomass management, activated biocarbons, chemical activation, conventional/microwave heating, ionic dyes removal, wastewater treatment

## Abstract

The main objective of the work was to prepare a series of new activated biocarbons by chemical activation of black chokeberry seed and to assess their suitability for removing cationic and anionic dyes from an aqueous medium. Activation of the precursor was performed at 550 °C with orthophosphoric acid, using conventional or microwave-assisted heating. The activated biocarbons were characterized in terms of elemental composition, textural parameters, surface morphology, acid-base character of the surface, as well as electrokinetic properties. Adsorption tests were carried out against two organic compounds: methylene blue (thiazine dye of cationic character) and Congo red (azo dye of anionic character). The influence of the initial dye concentration (5–120 mg/L), temperature (20–40 °C), and solution pH (2–10) on dye removal efficiency from the liquid phase was investigated. Additionally, kinetic adsorption tests were carried out to determine the rate and mechanism of the dyes removal process. Microwave-assisted chemical activation with H_3_PO_4_ proved to be a very effective approach for generating a high specific surface area (884 m^2^/g) and a micro/mesoporous structure, which directly increases the adsorption capacity of activated biocarbons towards cationic and anionic synthetic dyes. The maximum adsorption capacities for methylene blue and Congo red were 194.5 and 68.6 mg/g, respectively. It was also confirmed that the choice of heating method at the activation stage plays a key role in determining the physicochemical properties and adsorption performance of the activated biocarbons prepared from waste biomass. In general, carbonaceous adsorbents derived from black chokeberry seeds exhibit high potential for the treatment of dye-contaminated wastewater.

## 1. Introduction

The rapid development of industry, in addition to benefits such as making life easier and increasing its quality and comfort, also brings negative consequences in the form of natural environment degradation, including significant pollution of both groundwater and surface water. Discharging sewage into water (both industrial and municipal) poses a serious threat to the health and life of living organisms. Therefore, a significant challenge today is to remove a number of pollutants from water, such as organic dyes from the textile industry, which are generated in huge quantities every day [1]. Water purification (especially freshwater resources) is a very important issue nowadays, as their quantity is constantly decreasing due to adverse climate change. Moreover, according to WHO forecasts, by 2050 the amount of freshwater resources should increase by about 30% to meet the needs of the rapidly growing human population.

For the above reasons, research aimed at developing much more effective water treatment methods (including physical, chemical and biological approaches) is of great interest to scientists and researchers worldwide. Adsorption processes are very popular among water treatment methods due to their operational simplicity and relatively low cost. They involve the removal of pollutants using various kinds of adsorbents such as zeolites, molecular sieves, or a whole range of carbon materials, particularly activated biocarbons.

Activated biocarbons are carbon materials with a highly developed porous structure and a large specific surface area [2]. Thanks to this structure, they exhibit very good adsorption properties against most pollutants occurring in the liquid and gaseous phase. The prefix “bio-” results from the use of biomass as a precursor for their production. Activated biocarbons are currently obtained from plant materials such as wood waste [3], corncobs [4], fruit peels and pits [5], coconut shells [6] and other nuts [7], as well as from hay [8], straw [9], and even weeds (e.g., horsetail) [10]. Reducing the amount of waste biomass is highly desirable, as its decomposition releases large quantities of greenhouse gases. This makes the production of activated biocarbons relatively inexpensive, due to the low cost of the precursor acquisition, and at the same time highly beneficial for the environment, as it significantly reduces the volume of solid waste disposed of in landfills.

In order to produce carbonaceous adsorbents, biomass is subjected to an activation process. Activation can be carried out by physical and chemical methods. The typical physical process involves carbonization of biomass at a temperature of 500–900 °C in the absence of oxygen, followed by partial gasification of the obtained material (biochar) with an oxidizing agent, such as steam or carbon dioxide, at a temperature of 800–1000 °C. Carbonization eliminates elements other than carbon from the material, and activation with gases provides the desired porous structure [11]. The main advantages of this method are its relatively low cost and the use of non-aggressive chemical reagents.

Chemical activation, on the other hand, involves impregnating the precursor with an activating substance, which may be zinc chloride, orthophosphoric acid, potassium or sodium hydroxide, and potassium or sodium carbonate. The material prepared in this way is then subjected to pyrolysis at a temperature of 500–800 °C, in an inert gas atmosphere [12]. The advantages of this method include shorter activation time and the possibility of using lower temperatures during processing. Nevertheless, it is more expensive than physical activation. Due to the highly developed porous structure obtained during the activation process, chemically activated biocarbons are excellent adsorbents, capable of effectively binding various types of pollutants on their surface [13].

There are reports in the literature on the production and potential applications of activated carbons obtained via chemical activation of berry seeds. The materials obtained in this way are characterized by a well-developed porous structure and high adsorption capacity for various pollutants. For example, Bazan-Woźniak and Pietrzak [14] obtained activated carbon with a specific surface area of 1177 m^2^/g, capable of adsorbing approximately 278 mg/g of rhodamine B, by activating raspberry seeds with potassium carbonate. Carbons with a well-developed specific surface area (1139–1238 m^2^/g) were also obtained by chemical activation of grape seeds with potassium carbonate, potassium hydroxide [15,16], and phosphoric acid [17]. The adsorbents obtained in this way were characterized by high sorption capacities for organic pollutants such as the herbicide diuron (3-[3,4-(dichlorophenyl)-1,1-dimethylurea]) or the cationic dye methylene blue. Carbonaceous adsorbents with slightly less favorable textural parameters (specific surface area ranging from 243 to 979 m^2^/g) were obtained by chemical activation of strawberry seeds with acetic acid [18] as well as pomegranate seeds [19] and laurel fruit seeds with ZnCl_2_ [20]. These materials were characterized by quite good sorption capacities for dyes such as crystal violet and methylene blue, as well as chloride derivatives of phenoxyacids.

The main objective of the presented study was to produce activated biocarbons via chemical activation of black chokeberry (*Aronia melanocarpa*) seeds using H_3_PO_4_, with particular emphasis on the comparative evaluation of conventional and microwave-assisted heating. The microwave-assisted method was applied due to its ability to provide more uniform volumetric heating of the entire sample as well as its potential to improve porosity development and surface functionalization compared to the conventional method in the same operating conditions. In the subsequent stage, the comprehensive physicochemical characterization of the obtained carbon materials was performed. Their suitability for removing ionic dyes (cationic methylene blue and anionic Congo red) from the aqueous phase was also assessed. Analysis of the obtained textural, surface and electrokinetic parameters together with adsorption data allowed us to propose the most probable dye adsorption mechanisms, providing a better understanding of their interactions with the surface of the carbonaceous materials. This knowledge is essential for the rational design of new adsorbents and may contribute to the optimization of adsorption methods for separating these types of pollutants from the liquid phase. Furthermore, the study demonstrated the potential of waste biomass valorization, which can contribute to environmental protection as well as to the development of cost-effective and, most importantly, sustainable adsorbents.

Black chokeberry exhibits numerous biological properties beneficial to human health, including anti-diabetic, anti-infective, anti-cancer, and anti-obesity effects. This is due to their high content of phenolic compounds, such as anthocyanins, cyanidins, phenolic acids, proanthocyanins, triterpenoids, and their analogues, which exhibit strong antioxidant properties. Consequently, chokeberry-based products have a protective effect on the heart, liver, and nervous system, reduce metabolic syndrome, and prevent cancer [21]. For these reasons, chokeberry fruit is widely applied in food/beverage industries (juices, jams, wines, teas, supplements, functional food production) [22] and in pharmacy (both extracts and single compounds isolated from the berries, including cyanidin-3-O-galactoside, chlorogenic acid, quercetin, and ursolic acid) [23].

The intensive processing of this fruit produces significant amounts of waste, primarily seeds. A very effective approach to utilizing this waste biomass seems to be the preparation of carbonaceous materials that can be used as adsorbents of pollutants from the aqueous phase. Despite the growing interest in activated biocarbons from fruit seeds, only a few studies have investigated black chokeberry seeds as an adsorbent precursor, and none of them have systematically compared conventional and microwave-assisted chemical activation, which highlights the novelty and motivation of this work. For example, Kozdrach and Radulski [24] investigated the effect of adding biochar derived from the pyrolysis of chokeberry biomass at 500 and 700 °C to lubricants formulated with vegetable oil on their tribological and rheological characteristics. In turn, Kukowska et al. [25] prepared a series of chokeberry seed-based biochars and activated biocarbons via pyrolysis at 400 °C, as well as by physical or direct activation with carbon dioxide using conventional/microwave-assisted heating at 700 and 800 °C. The obtained materials were applied for the adsorption removal of metals (copper, cadmium), metalloids (arsenic, selenium), and polymers (bacterial exopolysaccharide, ionic polyacrylamides) from the aqueous phase.

## 2. Materials and Methods

### 2.1. Production of Activated Biocarbons

**Figure 1:**
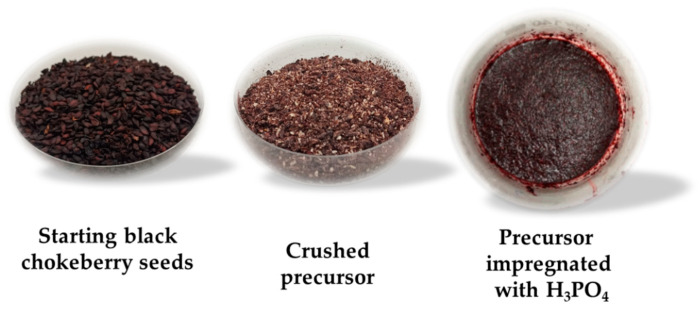
Pre-treatment of black chokeberry seeds.

Black chokeberry (*Aronia melanocarpa*) seeds (Figure 1) from Wielkopolska region (PPHU “GAMA” Sp. z o.o., Dęby Szlacheckie, Poland) were selected as a precursor for the production of carbonaceous adsorbents. The seeds were pre-dried at 40 °C and then crushed in a porcelain mortar to a grain size of <1.0 mm. The precursor prepared in this manner was impregnated with an 85% H_3_PO_4_ solution (EUROCHEM BGD, Tarnów, Poland), with the weight ratio of activator to precursor set as 2:1. After 12 h, the sample was dried to a constant mass at 110 °C in a laboratory oven (UFP 500 model, Memmert, Büchenbach, Germany), divided into two equal portions, and finally subjected to high-temperature treatment in a technical nitrogen atmosphere (flow rate 330 mL/min, Linde Gaz Polska, Kościan, Poland).

**Figure 2:**
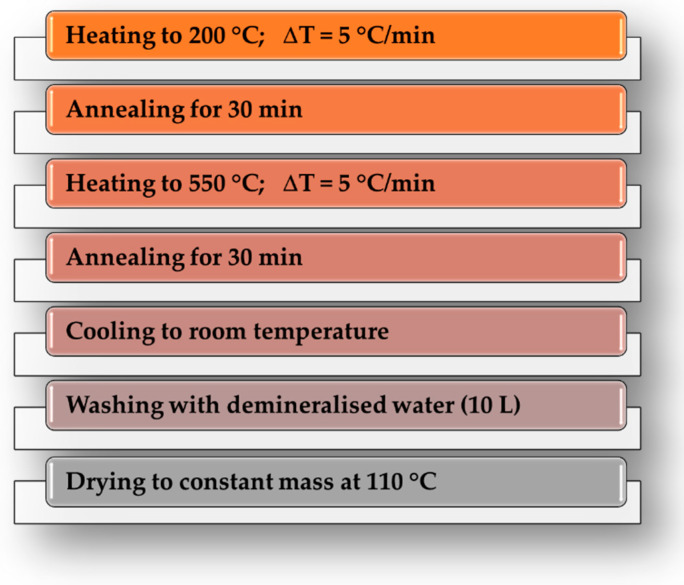
Schematic representation of the activated biocarbons production process.

One batch of material was placed in a nickel boat and heated in a conventional (C) laboratory furnace—model PRW75/LM with a single-heating-zone, equipped with a quartz tubular reactor with a length of 800 mm and an internal diameter of 70 mm (provided by Czylok, Jastrzębie-Zdrój, Poland). The second one was placed in a quartz crucible and heated in a microwave (M) muffle furnace (Phoenix model, CEM Corporation, Matthews, IL, USA). For both variants of heating, the procedure presented in Figure 2 was applied. The obtained materials were ground in a porcelain mortar to a grain size of approximately 0.1 mm and marked with the symbols AC and AM, respectively.

### 2.2. Characterization of Physicochemical Properties of Activated Biocarbons

Ash content for the starting black chokeberry seeds and the obtained activated biocarbons was determined according to the Polish Standard PN-ISO 1171:2002 [26]. The samples were burned in a microwave muffle furnace at 815 °C for 1 h, with each analysis performed twice.

The elemental composition of the raw material and the activated biocarbons was analyzed using a Vario EL III elemental analyzer (Elementar Analysensysteme GmbH, Langenselbold, Germany) to determine the levels of carbon, hydrogen, nitrogen, and sulfur. All analyses were performed in duplicate, and the repeatability of determinations for the reference substance was higher than 99.7%. The oxygen content was calculated by difference. Additionally, the chemical composition of starting black chokeberry seeds and both activated biocarbons was determined using the X-ray fluorescence method (ED-XRF Epsilon 5, PANalytical B.V., Almelo, The Netherlands).

The pH of the water extracts was measured using a pH-meter (model CP-401, equipped with a glass electrode EPS-1, ELMETRON, Zabrze, Poland). Powdered samples (0.5 g) were placed in conical flasks, and 50 mL of distilled water was added to each. The suspensions were magnetically stirred for 12 h until equilibrium was achieved, after which the pH was measured. The content of acidic and basic surface functional groups was determined by the Boehm method [27], which involves neutralization of surface species with HCl and NaOH. For this purpose, suspensions containing 0.25 g of carbonaceous material and 25 mL of 0.1 mol/L hydrochloric acid or sodium hydroxide (TARCHEM, Tarnowskie Góry, Poland) were prepared. After 24 h of shaking, the suspensions were filtered through a paper filter, and two 10 mL portions of the filtrate were titrated in the presence of 1% methyl orange solution (Avantor Performance Materials, Gliwice, Poland). All analyses were performed in duplicate.

The analysis of the types of functional groups present on the surface of the activated biocarbons was also performed using Fourier-transform infrared spectroscopy (Nicolet 8700 A, Thermo Scientific, Waltham, MA, USA).

The surface morphology of the carbonaceous materials was analyzed using a Quanta 250 FEG scanning electron microscope (FEI, Waltham, MA, USA).

The specific surface area and other textural parameters of activated biocarbons were calculated using the BET method based on the low-temperature nitrogen sorption isotherms measured at −196 °C with an ASAP 2020 apparatus (Micromeritics Instrument Corporation, Norcross, GA, USA). The total pore volume and average pore diameter were evaluated using the BJH method, while the surface area and micropore volume were calculated using the t-plot method.

### 2.3. Characterization of the Electrical Double Layer Formed on the Activated Biocarbons Surface

**Figure 3:**
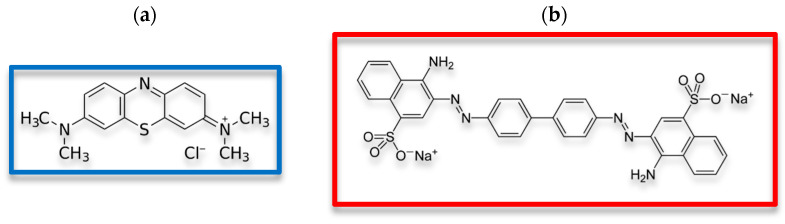
Structural formulas of methylene blue (**a**) and Congo red (**b**).

The dependencies of surface charge density (σ_0_) as a function of solution pH for both activated biocarbons without and with ionic dyes—cationic methylene blue (MB) and anionic Congo red (CR) (Chempur, Piekary Śląskie, Poland)—were determined using the potentiometric titration method. Additionally, the intersection of these curves with the x-axis enables the specification of the point of zero charge (pzc). This parameter is defined by the specific pH value at which the surface charge is equal to zero, i.e., the concentration of positively and negatively charged surface groups is the same. The titration process was controlled automatically using a Dosimat 765 microburette (Metrohm, Herisau, Switzerland) and a special computer program “Titr_v3” [28]. All experiments were performed in duplicate in the pH range of 3–11, for 50 mL suspensions containing 0.032 g of AC sample or 0.023 g of AM activated biocarbon, both without and with ionic dye at a concentration of 30 mg/L. The structural formulas of MB and CR are presented in Figure 3.

In analogous systems, the dependencies of zeta potential (ζ) as a function of solution pH for both activated biocarbons, without and with ionic dyes, were determined using the Doppler laser electrophoresis method. The intersection of these curves with the x-axis allows the determination of the isoelectric point (iep), which corresponds to the pH at which the total charge accumulated in the slipping plane area of the electrical double layer (edl) becomes zero, causing the solid particles to show a clear tendency to aggregate. The zeta (electrokinetic) potential was calculated using special software based on the Henry’s equation [29]. The electrophoretic mobility of activated biocarbon particles was measured using a Zetasizer Nano ZS 90 analyzer manufactured by Malvern Instruments (Malvern, UK). All experiments were performed in five parallel repetitions in the pH range 3–10, using 200 mL of suspensions containing 0.03 g of activated biocarbon, both without and with ionic dye at a concentration of 30 mg/L. Using the same equipment and the static light scattering method, the prepared suspensions were subjected to measurements of mean aggregate size (at pH 3, 6, and 9).

### 2.4. Characterization of the Adsorption Capacity of Activated Biocarbons Towards Ionic Dyes

Batch adsorption experiments using two different types of pollutants were conducted to evaluate the adsorption capacity of activated biocarbons derived from black chokeberry seeds. Cationic methylene blue (CAS No. 61-73-4) and anionic Congo red (CAS No. 573-58-0) were used as model adsorbates. The stock solutions (1000 mg/L) were prepared by dissolving 1.0 g of MB or CR in 1000 mL of distilled water, and then, by appropriate dilutions, working solutions of varying concentrations were obtained. The influence of temperature (20–40 °C) and solution pH (in the range 2–10) on the adsorption efficiency of both dyes was also investigated. In the case of the latter, the pH of the dye solutions was adjusted using 0.1 M HCl and NaOH solutions. The pH was measured using a CP-401 pH-meter equipped with a combined glass electrode. For the kinetics studies, the suspensions were shaken at 300 rpm for 6 h at a temperature of 25 °C and pH 6. Samples were collected at increasing time intervals: every 5 min (0–0.5 h), every 10 min (0.5–1.5 h), and every 30 min (1.5–6.0 h). All analyses were performed in duplicate.

For both organic dyes, the tests were carried out according to the following procedure: 25 mg of both activated biocarbons were added to a series of conical flasks and mixed with 50 mL of the dye solution. For Congo red, the initial concentration range was 5–70 mg/L, while for methylene blue it was 5–120 mg/L. The suspensions were shaken at 300 rpm at room temperature for 12 h to reach equilibrium (Unimax 1010 shaker equipped with an incubator (Heidolph Instruments GmbH & Co. KG, Schwabach, Germany)). The suspensions were then centrifuged for 15 min at 12500 rpm in a Frontier FC5515 centrifuge (OHAUS, Parsippany, NJ, USA) to minimize the presence of carbon particles in the analytes collected for spectroscopic analysis. CR and MB concentrations were determined using a Cary 100 Bio double beam UV–Vis spectrophotometer (Varian, Palo Alto, CA, USA), at wavelengths of 497 and 664 nm, respectively. Distilled water served as the reference. Quantification was performed based on previously prepared calibration curves.

The amounts of CR and MB adsorbed at equilibrium state (q_ad_, mg/g) were calculated from the Equation (1):

(1) $$q_{ad}=\frac{{∆C}_{dye}\cdot V_{sol}}{m}$$

where Δc_dye_—the difference between the initial and equilibrium organic dye concentration [mg/g], V_sol_—the volume of the adsorbate solution [L], m—the mass of the adsorbent [g].

The experimental adsorption data were analyzed by fitting them to the Langmuir, Freundlich, Temkin and Dubnin-Radushkevich isotherm models (Equations (2)–(5)):

(2) $$q_{e}=\frac{q_{m}K_{L}c_{e}}{1+K_{L}c_{e}}$$

(3) $$q_{e}=K_{F}c_{e}^{1/n}$$

(4) $$q_{e}=\frac{RT}{b_{T}}lnA_{T}+\frac{RT}{b_{T}}lnC_{e}$$

(5) $$q_{e}=q_{m}exp(-\beta \varepsilon^{2})$$

where q_e_—the amount of dye adsorbed per unit of adsorbent at equilibrium state [mg/g], q_m_—the maximum adsorption capacity [mg/g], K_L_—Langmuir isotherm constant [L/mg], c_e_—equilibrium concentration of dye [mg/L], K_F_—Freundlich isotherm constant [mg/g(mg/L)^1/n^], n—adsorption intensity, R—gas constant (8.314 J/(mol K), T—temperature [K], b_T_—Temkin constant [J/mol], A_T_—Temkin constant [L/g], β—Dubinin-Radushkievich constant, ε—adsorption potential [kJ/mol].

The kinetic experimental data were fitted using the pseudo-1st and the pseudo-2nd-order kinetic models, expressed by Equations (6) and (7):

(6) $$\frac{{dq}_{t}}{dt}=k_{1}(q_{e}-q_{t})$$

(7) $$\frac{{dq}_{t}}{dt}=k_{2}{\left(q_{e}-q_{t}\right)}^{2}$$

where q_e_—the amount of dye adsorbed in the equilibrium state [mg/g], q_t_—the amount of CR or MB adsorbed after time “t” [mg/g], k_1_—the pseudo-1st-order rate constant [1/min], k_2_—the rate constant of the pseudo-2nd-order adsorption [g/(mg·min)].

## 3. Results and Discussion

### 3.1. Physicochemical Properties of Activated Biocarbons

The data presented in Table 1 clearly indicate that both activation products are characterized by a much higher degree of carbon matrix ordering than the original black chokeberry seeds. The increase in C^daf^ content is much more pronounced in the case of the conventionally heated AC sample. The hydrogen content and levels of other heteroatoms vary significantly depending on the heating method used. Only the S^daf^ content in the structure of the obtained activated biocarbons remains at the same level. Moreover, both carbon materials are characterized by a significantly higher contribution of mineral admixtures (ash), which can act as ballast and negatively affect the physicochemical and sorption properties of carbonaceous adsorbents. In this respect, the AM sample heated with microwave energy performs slightly better. Such significant differences compared to the precursor confirm that both heating methods lead to distinct pyrolysis mechanisms. Conventional heating favors more uniform, slower thermal transformations, while microwaves promote selective, fast, and locally intense processes, resulting in different elemental profiles and final properties of activated biocarbons. The results obtained in this study are consistent with previous findings reported by Koczenasz et al. [30], who investigated H_3_PO_4_-activated biocarbons produced from sage stems and observed analogous changes in carbon ordering and heteroatom content. However, an inverse relationship was observed for the ash content.

Information on the elemental composition was supplemented by the XRF analysis results presented in Table 2. These data indicate that the structure of both activated bio-carbons derived from black chokeberry seeds also contains small amounts of elements characteristic of plant biomass, such as Si, K, Ca, Mn, and Cu. The increased phosphorus content (especially in the case of AM biocarbon) results from the use of orthophosphoric acid as an activating agent and its incorporation into the carbon matrix as phosphate or pyrophosphate groups [31,32]. A rather unusual situation was observed in the case of Fe, Ni, and Cr content. The increased levels of these elements in the AC sample, compared to the corresponding AM material obtained in a microwave furnace, suggest that the metal boats used during conventional heating may have been partially damaged by the combined action of acid and high temperature, leading to contamination of the carbonaceous material.

One of the important physicochemical parameters of activated biocarbons (especially from the adsorption point of view) is their surface chemistry. For this reason, in the next stage of the research, the acid-base properties of carbon materials obtained via thermal conversion of black chokeberry seeds were determined. As shown in Table 3, both biocarbons differ primarily in the content of acidic functional groups. The product of microwave-assisted activation has almost three times more functional groups of this type on its surface than the material obtained in a conventional furnace, which is probably related to the significantly higher oxygen content (Table 1). The more acidic nature of the AM sample is also confirmed by the pH value of its aqueous extract, which is below 4. Comparable acid-base surface properties have been reported for carbonaceous materials produced by chemical activation with phosphoric acid, e.g., from grape seeds [17], sage stems [30], jujube stones [33], and many different types of waste biomass [34].

Both materials also contain some surface functional groups of a basic character. Furthermore, in the AC sample, a slight predominance of these groups over the acidic ones is observed. However, the abundance of basic surface functional groups in phosphoric acid-activated biocarbons is considerably lower compared to that observed in materials prepared via one- or two-step physical activation of chokeberry seeds with CO_2_ [25], indicating a strong influence of activation method on the surface chemistry.

**Figure 4:**
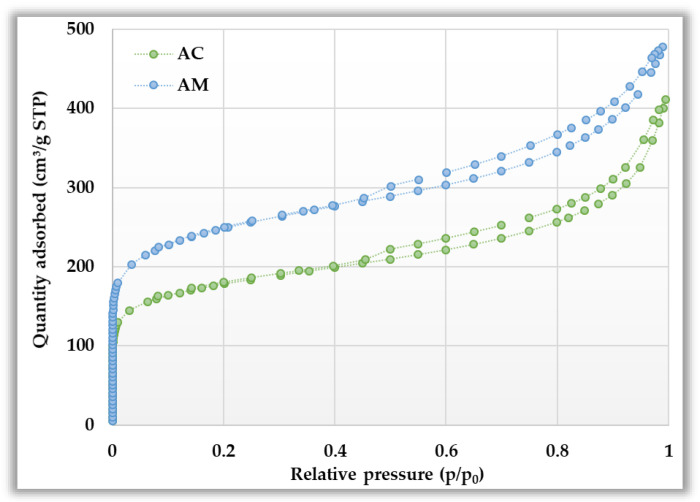

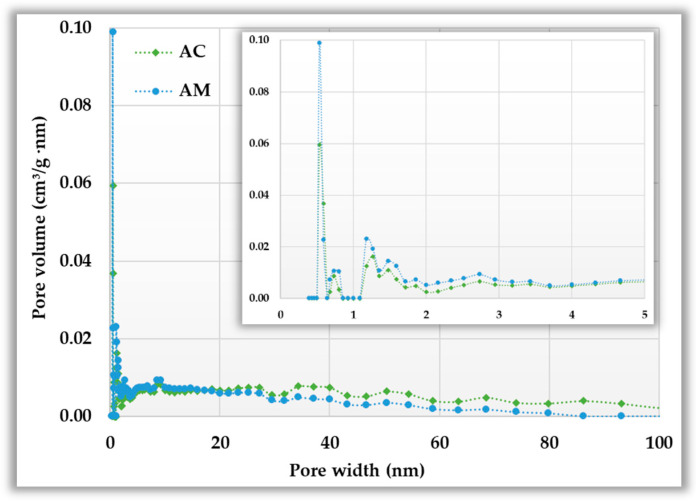
Low-temperature nitrogen adsorption/desorption isotherms (**top panel**) and pore size distribution (**bottom panel**) for activated biocarbons derived from black chokeberry seeds.

Textural properties, including surface area, pore volume, and pore size distribution, providing important information on the internal structure of activated biocarbons, are detailed in Table 4 and Figure 4. Based on these data, it can be concluded that chemical activation of black chokeberry seeds with orthophosphoric acid allows for quite effective development of the specific surface area and porous structure. However, microwave-assisted activation is more advantageous in this respect. The AM sample is characterized not only by a considerably higher BET specific surface area (~250 m^2^/g) but also by a higher contribution of micropores in the structure. Porosity analysis reveals that the structure of both activation products is dominated by mesopores and macropores, which typically promotes the effective adsorption of pollutants with large molecular sizes and facilitates the transport of the adsorbate into the interior of the adsorbent particles.

The analysis of the literature data indicates that chemical activation of chokeberry seeds with H_3_PO_4_ (especially in a microwave furnace) gives much better textural parameters than direct or physical activation of the same precursor with CO_2_, for which the specific surface area (S_BET_) ranged from 88 to 266 m^2^/g [25]. Unfortunately, this solution is not as effective as in the case of grape seeds (S_BET_ = 1139 m^2/^g) [17] and especially pumpkin seeds treated with orthophosphoric acid [35], where the surface area reached 1421 m^2^/g. It is therefore worth considering further optimization of the activated biocarbon production process, for example, by adjusting the ratio of reagents, heating time, or final temperature.

**Figure 5:**
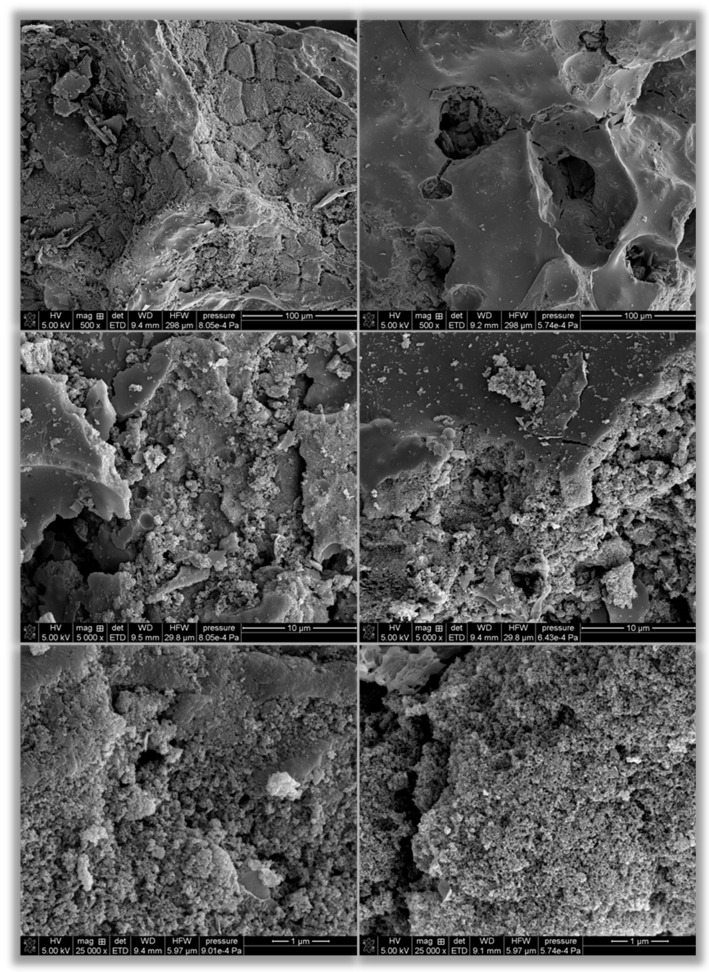
SEM images of activated biocarbons derived from black chokeberry seeds: AC (**left side**), AM (**right side**).

In the next step, the morphology of the produced carbonaceous adsorbents was examined. SEM images obtained at different magnifications are shown in Figure 5. At the lowest magnification (×500), significant differences are visible between the conventionally and microwave-heated samples. In the case of AC biocarbon (left side), the particle surface is clearly rough and cracked. Numerous grooves of varying thickness and light-colored fragments are also visible, which can be attributed to the presence of mineral phases, including the so-called phosphate film formed as a result of activation with orthophosphoric acid. In the case of AM biocarbon, the particle surface is smoother and covered with only a small amount of light and granular fragments. However, numerous pores of very different sizes, shapes, and depths are clearly visible. At magnifications of ×5000 and ×25,000, the differences between the two materials are less noticeable. Both materials are characterized by a developed and very rough microstructure composed of fine aggregates, numerous crevices, and irregular pores, suggesting significant destruction of the raw material’s structure during thermochemical treatment in the presence of H_3_PO_4_. The porous microstructure visible in the images confirms the results of textural analysis, which indicate the predominance of mesopores and macropores (the presence of micropores cannot be confirmed due to the limited resolution of SEM microscopy).

### 3.2. Electrical Double Layer Structure on the Activated Biocarbons Surface in the Ionic Dyes Presence

**Figure 6:**
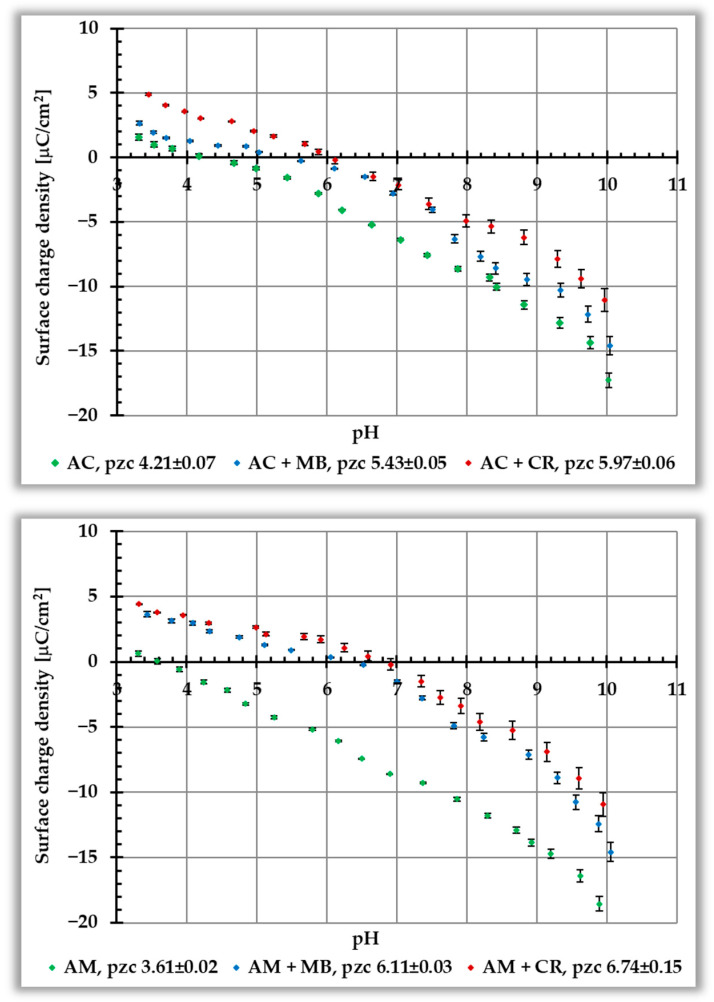
Dependence of surface charge density on solution pH for AC (**top panel**) and AM (**bottom panel**) activated biocarbons in the presence of ionic dyes.

The dependencies of the surface charge density (σ_0_) of activated biocarbons without and with ionic dyes, as a function of solution pH, are presented in Figure 6. They enable the determination of points of zero charge (pzc), which for the tested materials are 4.21 for AC and 3.61 for AM. Thus, both activated biocarbons are acidic in nature, and at pH values higher than pH_pzc_, their surfaces are negatively charged. In this pH range, the adsorption of cationic MB will be electrostatically favored. Nevertheless, both dyes are bound to the solid surface, as evidenced by changes in the surface charge (or more precisely, its increase in the entire pH range tested) in their presence. The adsorption of long CR molecules is rather perpendicular to the surface, due to the electrostatic repulsion. They probably bind at one end via a sulfate group, causing an increase in the surface charge of the carbonaceous material.

In the case of cationic MB, because of electrostatic attraction, their molecules adsorb more parallel to the surface. As a consequence, numerous positively charged groups of this dye (not directly bound) are present in the by-surface layer of the solution, causing an increase in the surface charge density. This interfacial behavior is accompanied by a shift of the pH_pzc_ points of systems containing organic substances towards higher pH values. As shown in Figure 6, the differences between σ_0_ curves obtained for systems with ionic dyes and those without adsorbates are more pronounced for the AM activated biocarbon. This is attributed to their higher adsorption on the carbonaceous material prepared using microwave-assisted heating.

**Figure 7:**
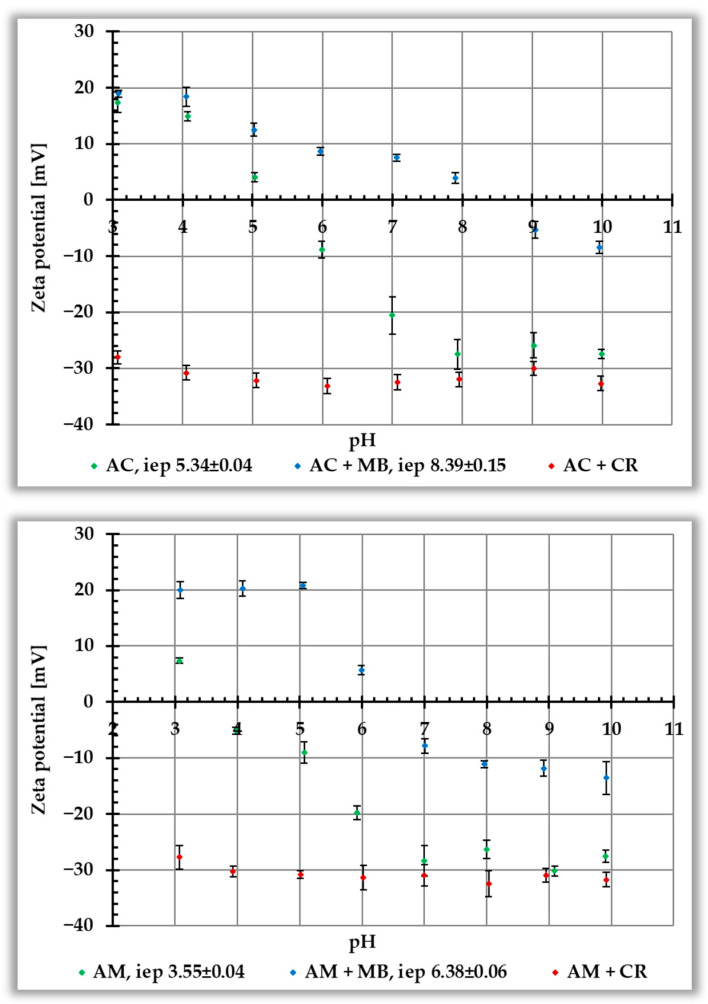
Dependence of zeta potential on solution pH for AC (**top panel**) and AM (**bottom panel**) activated biocarbons in the presence of ionic dyes.

Figure 7 shows the dependence of the zeta potential (ζ) of activated biocarbons without and with ionic dyes, as a function of solution pH. For carbonaceous materials without adsorbates, the isoelectric point (iep) values are 5.34 and 3.55 for AC and AM samples, respectively, falling within the acidic pH range. Adsorption of cationic MB causes a significant increase in electrokinetic potential in the entire pH range tested and a shift of the iep towards higher pH values, i.e., up to 8.39 for the AC sample and to 6.38 for the AM ones.

The situation is completely different in the case of anionic CR. In its presence, a rapid decrease in the zeta potential is observed—the ζ reaches a value of approximately −30 mV in the entire pH range studied. Therefore, it is not possible to determine the position of the iep in the presence of this dye. The main factor responsible for the observed changes is the presence of ionic groups of adsorbed organic molecules in the slipping plane area—positively charged in the case of MB (leading to an increase in zeta potential) and negatively charged in the case of CR (resulting in a decrease in zeta potential). Moreover, in the case of Congo red dye, which adsorbs more perpendicularly to the activated biocarbon surface, some contribution to the reduction in the electrokinetic potential may also come from the shift of the slipping plane (caused by adsorption layers with considerable thickness).

The analysis of the pH_pzc_ and pH_iep_ positions observed for the tested systems indicates that the corresponding values are lower in the case of the sample prepared via microwave heating. This confirms the more acidic surface nature of this material, which is consistent with the data presented in Table 3. Similar trends were reported by Koczenasz et al. [30], who used two heating variants (conventional and microwave) for the chemical activation of sage stems with H_3_PO_4_. In turn, research performed by Kukowska et al. [25] showed that carbonaceous materials obtained by direct or two-step physical activation of chokeberry seeds using carbon dioxide are characterized by pH_pzc_ in the alkaline range. Moreover, the pH_pzc_ of activated biocarbons obtained by microwave heating was higher than that of an analogous sample prepared in a conventional manner. This effect was particularly pronounced for materials activated at the higher temperature, i.e., at 800 °C.

**Figure 8:**
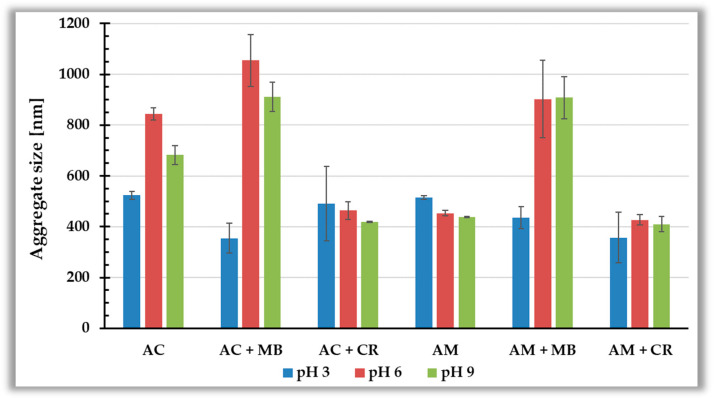
Mean aggregate sizes of activated biocarbons particles formed in the absence and presence of ionic dyes at pH 3, 6, and 9.

The average sizes of aggregates formed by the activated biocarbons particles in solution without and with ionic dyes at pH 3, 6, and 9 are shown in Figure 8. Analysis of these data indicates that in suspensions without dyes, AC particles form larger aggregates than the AM particles, with the greatest size observed for the AC system at pH 6. This is probably associated with the relatively low absolute value of zeta potential (around 10 mV), which does not provide effective electrostatic repulsion between the particles of this carbonaceous material. The addition of methylene blue to the colloidal suspension of both biocarbons causes an increase in the aggregate sizes in most of the studied systems, due to effective neutralization of the solid’s negative charge by the positively charged MB molecules. The exception is pH 3, at which the cationic dye and the solid surface have the same positive charge sign, and the most probable mechanism under such pH conditions is electrosteric repulsion. In turn, the adsorption of Congo red increases the stability of the tested suspension, leading to a decrease in the average aggregate size. The presence of thick, mutually repelling adsorption layers of this anionic dye leads to further enhancement in the stability of the studied colloidal dispersions.

### 3.3. Adsorption Performance of the Activated Biocarbons Toward Synthetic Dyes

To evaluate the adsorption capacity of the prepared activated biocarbons and assess their suitability for the removal of synthetic organic dyes with various ionic nature from aqueous solutions, methylene blue (thiazine dye of cationic character) and Congo red (azo dye of anionic character) were used as model adsorbates.

**Figure 9:**
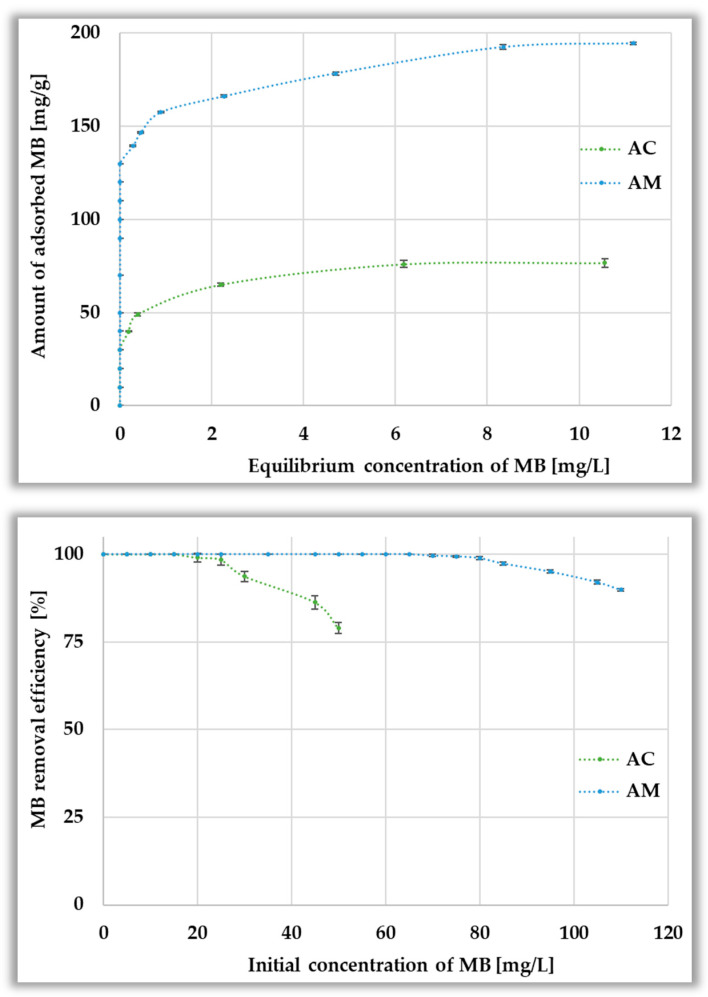
Equilibrium isotherms (**top panel**) and methylene blue adsorption efficiency from aqueous solution (**bottom panel**).

Figure 9 illustrates the equilibrium adsorption of methylene blue on the activated biocarbons and the corresponding removal efficiency from the aqueous phase. As observed, both isotherms exhibit an L-shape, suggesting minimal competition between water and methylene blue molecules for the active sites located on the surface of the activated biocarbons [36]. For both materials, the amount of methylene blue adsorbed at equilibrium increases with increasing initial dye concentration until saturation is reached. The microwave-assisted activation product (AM) proved to be a significantly more effective adsorbent for the cationic dye (194.52 ± 0.75 mg/g), exhibiting a sorption capacity almost 2.5 times higher than the sample prepared by heating in a conventional furnace (AC, 76.72 ± 2.37 mg/g). Moreover, the AM biocarbon allows for 100% removal of methylene blue over a wide range of initial concentrations (5–65 mg/L), while the AC sample does not completely adsorb the dye even at an initial concentration of 20 mg/L. The significantly better adsorption properties of the microwave-activated sample are most probably the result of a larger specific surface area, a higher content of acidic functional groups on its surface, and a lower pH_pzc_ value, which enhances interactions with the cationic dye.

**Figure 10:**
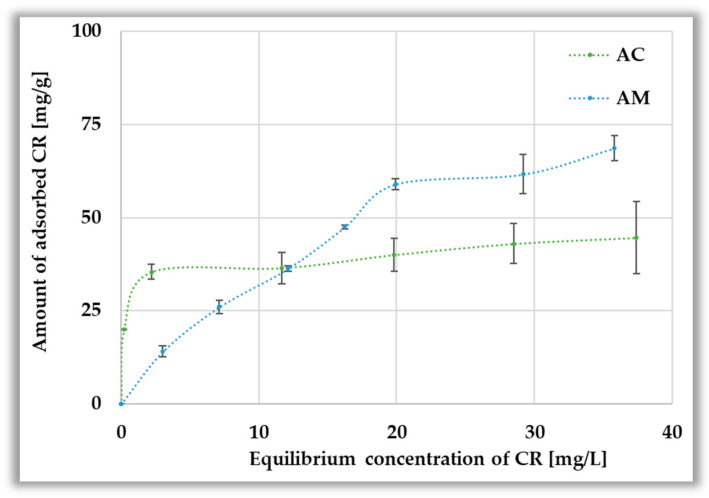

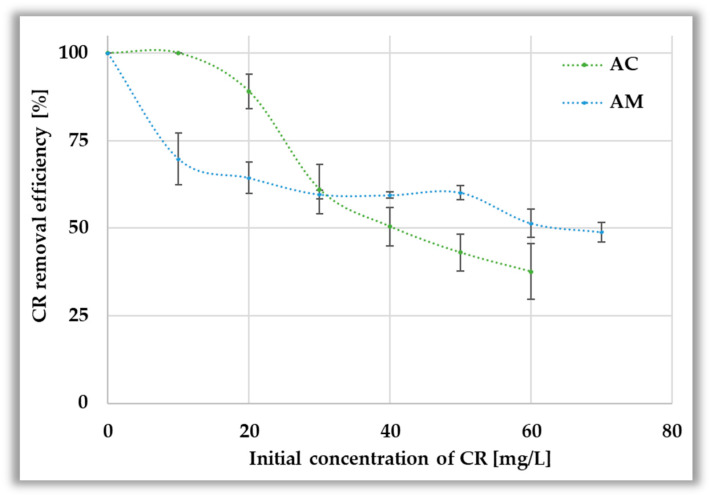
Equilibrium isotherms (**top panel**) and Congo red adsorption efficiency from aqueous solution (**bottom panel**).

According to the data presented in Figure 10, the adsorption of the anionic dye is not as efficient as that of methylene blue. This is evidenced by both significantly lower maximum sorption capacities (44.79 ± 9.75 and 68.4 ± 3.43 mg per g of AC and AM samples, respectively), as well as lower dye removal efficiency observed already at concentrations of 10–20 mg/L. The lower adsorption capacity is most probably a consequence of the larger size of Congo red molecules, which makes it difficult for them to penetrate into pores, as well as the electrostatic repulsion between the anionic dye molecules and the negatively charged surface of activated biocarbons, which may limit the possibility of creating strong sorption interactions.

Table 5 presents the results of fitting the experimental data to the Langmuir, Freundlich, Temkin, and Dubinin-Radushkevich isotherm models, including the calculated parameters and correlation coefficients. Analysis of these data indicates that the adsorption of MB on both carbonaceous materials obtained from black chokeberry seeds most likely proceeds according to the Langmuir mechanism, i.e., in the form of a monolayer coating of the biocarbon surface with dye molecules. This is evidenced by the very high values of the fit coefficient R^2^, which are 0.9986 and 0.9984, respectively. Moreover, the maximum adsorption capacities of the monolayer (q_m_) determined from the Langmuir model were 76.9 mg/g and 192.3 mg/g, respectively, which are very close to the equilibrium values q_e_ determined experimentally. High values of the K_L_ constant (9.28 and 17.33 L/mg) may indicate a strong affinity of methylene blue molecules for the surfaces of both activated biocarbons; however, the AM sample appears to exhibit a stronger interaction with this adsorbate. The Freundlich model also fits the experimental data well, particularly for the microwave-activated sample, for which the R^2^ value is 0.9918. This observation suggests that the surface of the obtained adsorbents exhibits significant heterogeneity. In turn, very low values of the 1/n exponent, especially in the case of the AM biocarbon (0.09), may indicate favorable adsorption conditions and the presence of many sites with high adsorption energy, which is quite typical for materials rich in micropores and having a very heterogeneous surface.

The data presented in Table 5 confirm the less favorable sorption capacity of activated biocarbons towards Congo red, which indicates that the properties of the adsorbate significantly influence the mechanism and intensity of adsorption. In the case of the conventionally heated sample, a better fit to the experimental data was obtained for the Langmuir model—R^2^ = 0.9948. However, the q_m_ and K_L_ parameters are considerably lower than those for the previously discussed methylene blue, which may indicate the weaker affinity of the anionic dye to the surface of activated biocarbons. In the case of the Freundlich model, the correlation coefficient is significantly lower, reaching only 0.7896. However, the very low value of the 1/n parameter may suggest that high-energy active sites occur on the surface of this adsorbent, although they are utilized to a lesser extent by CR molecules. In the case of the second adsorbent, the situation is opposite. A much better fit to the experimental data was obtained using the Freundlich model (R^2^ = 0.9822), while for the Langmuir model, it was only 0.8136, suggesting a more heterogeneous nature of the AM sample surface. A low K_L_ value and a moderate 1/n value indicate less favorable sorption conditions, which may result, among others, from weaker electrostatic interactions between the adsorbent surface and Congo red molecules, endowed with a charge of the same sign. The above relationships suggest that the mechanism of dye adsorption on the surface of activated biocarbons obtained from black chokeberry seeds is probably determined by the structure and chemical properties of the adsorbed organic compound. The other two isotherm models used in this study provided only limited insight into the interactions between MB/CR molecules and carbonaceous adsorbents. The bT parameter calculated from the Temkin model is higher for the systems containing the AC sample compared to those with the AM material, which may indicate that both dyes bind more strongly to the surface of activated biocarbon obtained by conventional heating. The adsorption energy calculated from the Dubinin–Radushkevich model suggests that methylene blue is most likely adsorbed on the surfaces of both carbonaceous materials mainly via chemisorption, as the ε value exceeds 20 kJ/mol in both cases. In contrast, the adsorption of Congo red is more variable: for the AC sample, ε is about 29 kJ/mol, indicating chemisorption, whereas for the AM sample, ε does not exceed 10 kJ/mol, suggesting that physisorption is the dominant mechanism [37]. However, further research is needed to clarify this issue. Analysis of the individual parameters of the Temkin and Dubinin–Radushkevich models indicates that, despite relatively high values of the R^2^ fit coefficients, these models do not fully reflect the course of experimental adsorption.

**Figure 11:**
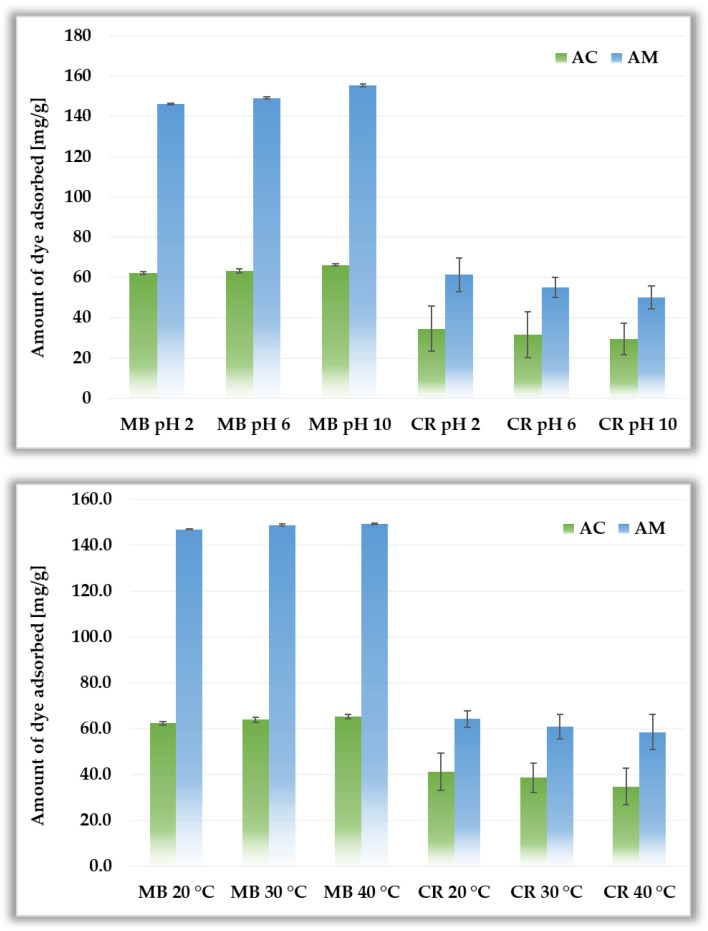
Influence of pH (**top panel**) and system temperature (**bottom panel**) on methylene blue and Congo red adsorption on activated biocarbons derived from black chokeberry seeds.

Figure 11 presents the changes in the adsorption efficiency of MB and CR as a function of the system pH and temperature. In the case of methylene blue, both activated biocarbons exhibit the highest adsorption efficiency at pH 10. This is most likely a consequence of the fact that at higher pH values (pH > pH_pzc_), the surface functional groups are deprotonated, which causes the adsorbent surface to become negatively charged and therefore interact more easily with cationic dye molecules [38]. In the case of anionic dye, the opposite trend is observed, i.e., the highest sorption capacity is achieved at pH 2 (below the pH_pzc_ of both adsorbents), when the surface is positively charged, which favors electrostatic attraction with CR molecules [39].

The effect of system temperature on adsorption efficiency is also diverse depending on the adsorbate used. The sorption capacity of the AC and AM toward methylene blue increases slightly (by ~2–3 mg/g) as the solution temperature increases from 20 °C to 40 °C. In the case of Congo red, the differences are slightly greater and reach approximately 6–7 mg/g, however the highest efficiency of CR removal is observed at 20 °C. At higher temperatures, the mobility of adsorbate molecules increases, facilitating their diffusion into the pores of activated biocarbons. In addition, the negatively charged biocarbon surface electrostatically attracts the dye cations. For smaller MB molecules, this may result in greater sorption capacity. In the case of an anionic dye such as CR, these favourable electrostatic interactions do not occur, and the considerably larger size of CR molecules may limit their access to the internal pore structure. Nevertheless, it should be clearly noted that the effect of solution pH and system temperature on the adsorption capacity of the biocarbons derived from black chokeberry seeds (particularly in the case of MB) is less significant than the impact of the heating variant applied during the activation procedure.

**Figure 12:**
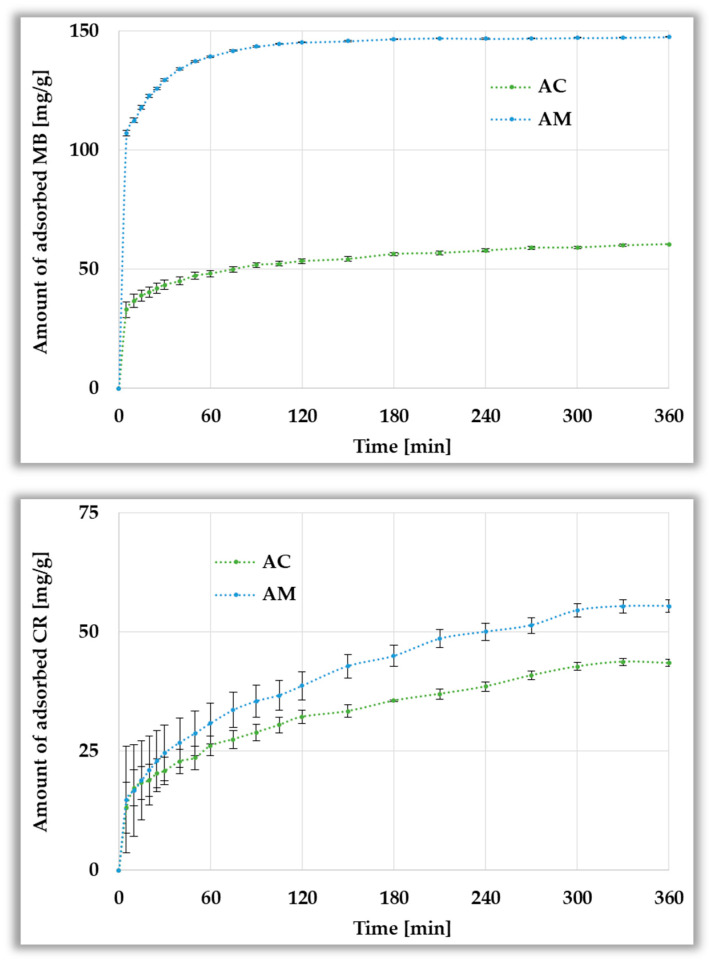
Influence of contact time on the adsorption of methylene blue (**top panel**) and Congo red (**bottom panel**) on activated biocarbons derived from black chokeberry seeds.

Figure 12 illustrates the effect of the contact time of the activated biocarbons with adsorbate molecules on the obtained sorption capacities. The adsorption behavior of AC and AM biocarbons toward methylene blue and Congo red exhibits different trends. As the process progresses over time, the efficiency of MB and CR removal increases; however, pronounced differences in adsorption intensity are observed between the two dyes. The adsorption rate of the cationic dye is very high for the first 60 min, then the process gradually slows down, and after about 180 min, the adsorption equilibrium is reached, which is attractive from a practical point of view. In the case of the anionic Congo red molecules, the intensity of the adsorption process is much lower, as a result of which the equilibrium state is reached only after about 300 min. The observed differences in adsorption behavior can be attributed to variations in the molecular sizes of these two dyes and, to some extent, their chemical properties, such as surface charge. The smaller size of methylene blue molecules allows them to diffuse more easily through the pores of the carbon matrix [40,41]. In this context, the role of electrostatic interactions between MB molecules and the functional groups present on the adsorbent surface cannot be ignored [41].

**Figure 13:**
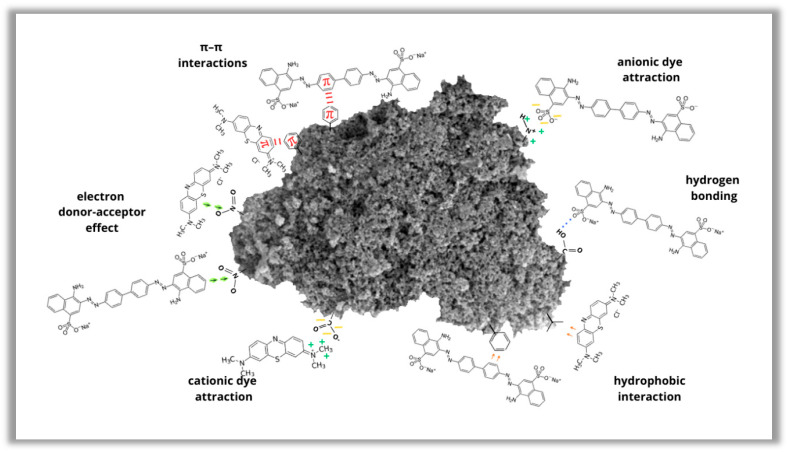
Probable adsorption mechanism of methylene blue and Congo red on the surface of activated biocarbon.

Kinetic data for the adsorption of cationic and anionic dyes were fitted using pseudo-first-order (PFO) and pseudo-second-order (PSO) models. As shown in Table 6, the PFO model underestimates the adsorption capacity for methylene blue, indicating that it does not adequately describe the adsorption mechanism. In contrast, the PSO model provides a much better fit to the experimental data, with coefficients of determination R^2^ very close to unity. This suggests that the adsorption of MB molecules is predominantly governed via chemical interactions (chemisorption), such as electrostatic attraction or electron exchange between the adsorbent and the adsorbate [42,43], which is consistent with the equilibrium adsorption results discussed earlier. In the case of Congo red, a better fit of the experimental data to the pseudo-second-order model is also observed. The q_cal_ values determined by PSO are closer to q_e_ than those obtained from the PFO model, even though the R^2^ coefficients for both models are comparable. The differences between the two models are not as pronounced as in the case of methylene blue; however, the PSO model better describes the CR adsorption kinetics, suggesting the involvement of chemical processes and the possible influence of surface interactions of various character (for example, hydrogen bonds or π–π interactions). A schematic representation of the possible interactions between the dye molecules and the activated biocarbons surface is presented in Figure 13.

**Figure 14:**
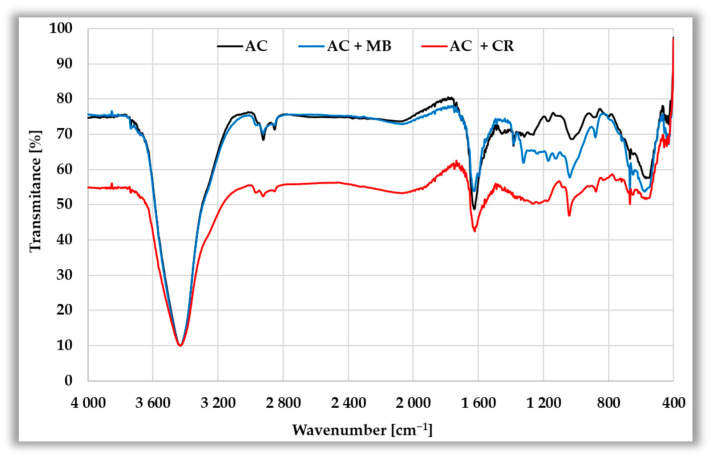

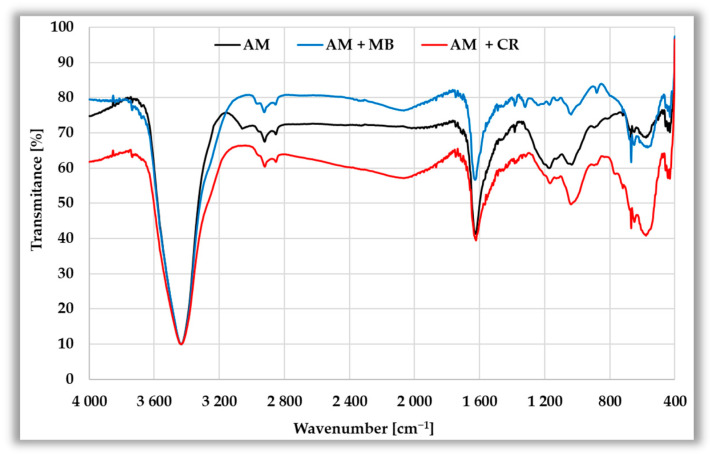
FT-IR spectra of the activated biocarbons before and after adsorption of methylene blue and Congo red.

Figure 14 shows the FTIR spectra of the initial activated biocarbons and the materials after adsorption of methylene blue and Congo red. Overall, the spectra are very similar, indicating that the structure of the carbonaceous matrix remains unchanged. However, some differences in intensity and slight shifts of specific bands may suggest interactions between the dye molecules and functional groups present on the adsorbent surface. A broad and very intense band with a minimum around 3430 cm^−1^ present in all samples can be attributed to stretching vibrations of the O–H groups (surface hydroxyls or adsorbed water). After adsorption of dyes, especially Congo red, a significant decrease in transmittance is observed, which may indicate hydrogen interactions between this dye and the surface of AC and AM biocarbons [44]. Two less intense bands in the range of 2950–2850 cm^−1^ may be assigned to the C-H stretching vibrations of the aliphatic CH_3_ and CH_2_ groups [45]. Their low intensity may indicate a high degree of aromatization in the activated carbon structure. Analysis of the spectra indicates that adsorption of both dyes does not significantly affect the intensity of these bands. A distinct band with a minimum around 1630 cm^−1^ can be attributed to C=C vibrations of aromatic structures, as well as carbonyl groups (C=O). The changes in the width and intensity of this band, especially after CR adsorption, may indicate π–π interactions between the aromatic rings of the dye and the activated biocarbon structure [46]. In the case of the AC sample, additional bands are observed at approximately 1380 cm^−1^ and 1030 cm^−1^, which can be attributed to C–H bending vibrations of CH_2_ and CH_3_ groups and to stretching vibrations of C–O and C–O–C bonds, respectively. The band at ~1380 cm^−1^ is not detected for AM biocarbon; instead, a band with a minimum around 1171 cm^−1^ is observed, which is also associated with C–O stretching vibrations of oxygen-containing functional groups. After the adsorption of both dyes, changes in the intensity of the mentioned bands and slight shifts are observed, suggesting the involvement of these functional groups in interactions with methylene blue and Congo red molecules. In turn, the bands appearing in the region of 700–400 cm^−1^ can be attributed to the presence of mineral matter in the structure of the activated biocarbons.

### 3.4. Adsorption of Methylene Blue and Congo Red on Plant-Derived Activated Biocarbons

Table 7 compares the adsorption capacity of activated biocarbons derived from black chokeberry seeds towards methylene blue and Congo red, with other carbonaceous adsorbents obtained via chemical activation and described in the literature by other research groups. These data indicate that the obtained carbonaceous adsorbents, particularly the AM sample, show quite favourable results compared to other materials produced via chemical activation of different types of waste biomass [30,36,41,46,47,48,49,50,51,52,53,54,55,56].

In the case of methylene blue removal from aqueous solution, the black chokeberry seeds-based activated biocarbon produced via microwave heating shows quite high efficiency (194.5 mg/g). Nevertheless, other microwave-treated carbonaceous materials prepared, for example, from rubber seed pericarp using orthophosphoric acid activation [49] or from almond shells applying zinc chloride as the activator [51] are characterized by a very high sorption capacity towards MB, reaching values 348 and 314 mg/g, respectively. The microwave-assisted variant of sage stems activation with H_3_PO_4_ proved to be equally effective in obtaining an efficient adsorbent of cationic dye (almost 300 mg/g) [30].

The method of heating black chokeberry seeds during the preparation of activated biocarbons has less influence on the amount of Congo red adsorbed on the resulting material’s surface. The obtained values change in the range 45–69 mg/g and are rather low in comparison to other chemically activated sorbents. For example, activated biocarbon derived from tomato paste waste, synthesized through chemical activation with ZnCl_2_ and conventional heating, shows a very high CR adsorption efficiency of 435 mg/g [53]. An interesting approach was presented by Zubir and Zaini [41], who used a mixed H_3_PO_4_/ZnCl_2_ activating agent for chemical activation of *Pterocarpus indicus* twig biomass, resulting in a carbonaceous adsorbent with a Congo red adsorption capacity of 217 mg/g. Carbonaceous material obtained via ZnCl_2_ activation of red pumpkin skin also showed promising potential for the removal of anionic dye—140 mg/g [54]. In contrast, chemical activation of other activated biocarbons precursors, such as *Spathodea campanulata* flowers [46], jujube seeds [55], or waste black cardamom peels [56] under conventional heating conditions, led to the obtaining of less effective CR adsorbents.

## 4. Conclusions

It has been proven that agricultural waste, such as black chokeberry seeds, can be effectively utilized as a precursor for the production of relatively inexpensive and environmentally friendly adsorbents. The study has also demonstrated that the physicochemical properties and adsorption performance of H_3_PO_4_-activated biocarbons are strongly influenced by the heating method applied during synthesis. Orthophosphoric acid proved to be a highly reactive activating agent for black chokeberry seeds, allowing the production of carbonaceous materials with a well-developed specific surface area at a temperature of 550 °C, which is attractive from both an economic and environmental point of view. Moreover, the combination of orthophosphoric acid activation with microwave-assisted heating facilitates the synthesis of adsorbents with a hierarchical porous structure rich in mesopores, which may promote the effective removal of organic dyes differing in molecular sizes and ionic nature from aqueous solutions. Therefore, further research should be directed towards optimizing the production of activated biocarbons, particularly with respect to the influence of activation time and the precursor-to-activator ratio on their textural, surface, and electrokinetic properties, and consequently on the adsorption efficiency, especially for anionic dyes such as Congo red.

## Figures and Tables

**Table 1:** Ash content and elemental composition of the starting black chokeberry seeds and activated biocarbons obtained as a result of their thermochemical treatment.

Sample	Ash Content [wt. %]	C^daf 1,2^ [wt. %]	H^daf^ [wt. %]	N^daf^ [wt. %]	S^daf^ [wt. %]	O^diff 3^ [wt. %]
black chokeberry seeds	2.3 ± 0.2	52.3	6.8	2.3	1.2	37.4
AC	13.3 ± 0.5	86.3	2.7	2.8	0.1	8.1
AM	11.6 ± 0.4	77.2	1.7	3.5	0.1	17.5

^1^ daf—expressed on a dry and ash-free basis; ^2^ method error ≤ 0.3%; ^3^ diff—calculated by difference.

**Table 2:** XRF data regarding the chemical composition of activated biocarbons.

Element ^1^	Content [mg/g]		
	Chokeberry Seeds	AC	AM
Si	0.068 ± 0.006	0.007 ± 0.001	0.307 ± 0.023
P	0.194 ± 0.010	0.549 ± 0.047	3.441 ± 0.383
S	0.109 ± 0.017	0.035 ± 0.032	0.304 ± 0.031
Cl	0.010 ± 0.008	0.003 ± 0.002	0.139 ± 0.027
K	0.934 ± 0.011	0.001 ± 0.001	0.041 ± 0.005
Ca	1.150 ± 0.018	0.015 ± 0.012	0.526 ± 0.093
Cr	-	0.140 ± 0.108	0.004 ± 0.001
Mn	0.043 ± 0.007	0.007 ± 0.005	0.001 ± 0.001
Fe	0.082 ± 0.011	0.291 ± 0.208	0.099 ± 0.015
Ni	0.002 ± 0.001	0.154 ± 0.102	0.011 ± 0.002
Cu	0.015 ± 0.008	0.011 ± 0.006	0.061 ± 0.008

^1^ trace amounts of Zn, As, Mo, Zr, Sn, Ba, Pb (<0.1 mg/g).

**Table 3:** The acid-base properties of the carbonaceous adsorbents obtained via chemical activation of black chokeberry seeds.

Sample	pH of Water Extracts	Acidic Groups Content [mmol/g]	Basic Groups Content [mmol/g]	The Total Content of Functional Groups [mmol/g]
AC	4.30 ± 0.03	0.69 ± 0.04	0.85 ± 0.05	1.55 ± 0.09
AM	3.81 ± 0.06	1.87 ± 0.03	0.59 ± 0.03	2.46 ± 0.06

**Table 4:** Textural parameters of the carbonaceous adsorbents obtained via chemical activation of black chokeberry seeds.

Sample	Total		Micropore		Micropore Contribution [%]	Mean Pore Size [nm]
	Surface Area [m^2^/g] ^1^	Pore Volume [cm^3^/g]	Area [m^2^/g]	Volume [cm^3^/g]		
AC	635	0.637	291	0.127	19.9	4.02
AM	884	0.739	409	0.178	24.1	3.34

^1^ Method error 2–5%.

**Table 5:** Langmuir, Freundlich, Temkin, and Dubinin-Radushkevich isotherms parameters for the adsorption of methylene blue and Congo red on the activated biocarbons derived from black chokeberry seeds.

Model		Methylene Blue		Congo Red	
		AC	AM	AC	AM
**q_e_**		76.7	194.5	44.8	68.4
**Langmuir**	**q_m_**	76.9	192.3	44.2	88.5
	**K_L_**	9.28	17.33	1.30	0.08
	**R^2^**	0.9986	0.9984	0.9948	0.8136
**Freundlich**	**K_F_**	54.95	156.86	32.28	6.83
	**1/n**	0.16	0.09	0.08	0.68
	**R^2^**	0.9709	0.9918	0.7896	0.9822
**Temkin**	**b_T_**	256.9	162.9	781.3	100.7
	**A_T_**	401.5	38289.9	26208.8	0.5
	**R^2^**	0.9868	0.9880	0.7753	0.9514
**Dubinin-Radushkevich**	**β**	1.13 × 10^−9^	6.26 × 10^−10^	5.78 × 10^−10^	5.17 × 10^−9^
	**ε**	21.0	28.3	29.4	9.8
	**q_m_**	163.6	286.3	59.6	1524.0
	**R^2^**	0.9812	0.9892	0.7625	0.9859

q_e_—the amount of dye adsorbed per unit of adsorbent at equilibrium state [mg/g], q_m_—the maximum monolayer adsorption capacity [mg/g], K_L_—the Langmuir isotherm constant [L/mg], R^2^—the correlation coefficient, K_F_—the Freundlich isotherm constant [mg/g(mg/L)^1/n^], n—the adsorption intensity, b_T_—Temkin constant [J/mol], A_t_—Temkin constant [L/g], β—Dubinin-Radushkevich constant, ε—adsorption potential [kJ/mol].

**Table 6:** Parameters of kinetic isotherm models for methylene blue and Congo red adsorption.

Sample	q_e_	Pseudo-First-Order			Pseudo-Second-Order		
		q_cal_	k_1_	R^2^	q_cal_	k_2_	R^2^
**Methylene blue**							
AC	60.59 ± 0.04	24.83	0.01036	0.9772	61.73	0.00120	0.9985
AM	147.59 ± 0.27	25.07	0.01566	0.9375	149.25	0.00179	0.9999
**Congo red**							
AC	43.50 ± 0.32	37.06	0.01151	0.8700	39.53	0.00031	0.9767
AM	57.49 ± 0.71	45.55	0.00829	0.9735	61.35	0.00033	0.9811

q_e_—experimental adsorption capacity [mg/g], q_cal_—calculated adsorption capacity [mg/g], k_1_—the pseudo-first-order adsorption rate constant [1/min], k_2_—the pseudo-second-order adsorption rate constant [g/(mg∙min)], R^2^—the determination coefficient.

**Table 7:** Comparison of adsorption capacities of biomass-derived activated biocarbons towards methylene blue and Congo red.

Carbonaceous Adsorbent	Preparation Procedure	Maximal Adsorbed Amount [mg/g]	Reference
**Methylene Blue**			
Black chokeberry seeds derived activated biocarbon	Chemical activation with H_3_PO_4_ (conventional heating)	76.7	This study
Black chokeberry seeds derived activated biocarbon	Chemical activation with H_3_PO_4_ (microwave heating)	194.5	This study
Sage stems derived activated biocarbon	Chemical activation with H_3_PO_4_ (microwave heating)	298.4	[30]
Banana peel-derived activated biocarbon	Chemical activation with H_3_PO_4_ (conventional heating)	52.6	[36]
Pistachio nutshell-derived activated biocarbon	Chemical activation with H_3_PO_4_ (conventional heating)	183.1	[47]
Walnut shell-derived activated biocarbon	Chemical activation with H_3_PO_4_ (conventional heating)	247.1	[48]
Rubber seed pericarp-derived activated biocarbon	Chemical activation with H_3_PO_4_ (microwave heating)	347.8	[49]
Bamboo waste-derived activated biocarbon	Chemical activation with K_2_CO_3_ (microwave heating)	85.6	[50]
Almond shell-derived activated biocarbon	Chemical activation with ZnCl_2_ (microwave heating)	314.2	[51]
Sugarcane bagasse waste-derived activated biocarbon	Chemical activation with KOH (conventional heating)	136.5	[52]
**Congo Red**			
Black chokeberry seed-derived activated biocarbon	Chemical activation with H_3_PO_4_ (conventional heating)	44.8	This study
Black chokeberry seed-derived activated biocarbon	Chemical activation with H_3_PO_4_ (microwave heating)	68.4	This study
*Pterocarpus indicus* twig-derived activated biocarbons	Chemical activation with H_3_PO_4_/ZnCl_2_ (conventional heating)	217.0	[41]
*Spathodea campanulata* flower-derived activated biocarbon	Chemical activation with H_3_PO_4_ (conventional heating)	59.3	[46]
Tomato paste waste-derived activated biocarbon	Chemical activation with ZnCl_2_ (conventional heating)	435.0	[53]
Red pumpkin skin-derived activated biocarbon	Chemical activation with ZnCl_2_ (conventional heating)	140.2	[54]
Jujube seed-derived activated biocarbon	Chemical activation with H_3_PO_4_ (conventional heating)	9.8	[55]
Waste black cardamom peel-derived activated biocarbon	Chemical activation with KOH (conventional heating)	69.9	[56]

## Data Availability

The original contributions presented in this study are included in the article. Further inquiries can be directed to the corresponding authors.
